# Glioblastoma-specific anti-TUFM nanobody for *in-vitro* immunoimaging and cancer stem cell targeting

**DOI:** 10.18632/oncotarget.24629

**Published:** 2018-04-03

**Authors:** Neja Samec, Ivana Jovcevska, Jure Stojan, Alja Zottel, Mirjana Liovic, Michael P. Myers, Serge Muyldermans, Jernej Šribar, Igor Križaj, Radovan Komel

**Affiliations:** ^1^ Medical Centre for Molecular Biology, Institute of Biochemistry, Faculty of Medicine, University of Ljubljana, Ljubljana, Slovenia; ^2^ International Centre for Genetic Engineering and Biotechnology (ICGEB), Trieste, Italy; ^3^ Cellular and Molecular Immunology, Bioengineering Sciences Department, Vrije Universiteit Brussel, Brussels, Belgium; ^4^ Department of Molecular and Biomedical Sciences, Jožef Stefan Institute, Ljubljana, Slovenia

**Keywords:** glioblastoma multiforme, biomarkers, nanobodies, cytotoxicity, TUFM

## Abstract

Glioblastoma multiforme (GBM) is the most common and lethal form of brain tumor. The prognosis for patients remains poor, despite the combination of new preoperative and intraoperative neuroimaging, radical surgery, and recent advances in radiotherapy and chemotherapy. To improve GBM therapy and patient outcome, sustained drug delivery to glioma cells is needed, while minimizing toxicity to adjacent neurons and glia cells. This might be achieved through an anti-proteomic approach based on nanobodies, the single-domain antigen-binding fragments of heavy-chain antibodies of the camelid adaptive immune system. We report here on the validation and quantification of a nanobody raised against mitochondrial translation elongation factor (TUFM). Differential expression of TUFM was examined in different GBM cell lines and GBM tissue at the protein and mRNA levels, as compared to their expression in neural stem cells and normal brain tissue. We further used *in-silico* modelling and immunocytochemistry to define the specificity of anti-TUFM nanobody (Nb206) towards GBM stem cells, as compared to GBM cell lines (U251MG and U87MG cells). Due to its specificity and pronounced inhibitory effect on GBM stem cell growth, we propose the use of this anti-TUFM nanobody for GBM *in vitro* immunoimaging and potentially also cancer stem cell targeting.

## INTRODUCTION

Glioblastoma multiforme (GBM) is the most common primary malignant brain tumor, with an annual incidence of 5.26 per 100,000 people, corresponding to about 17,000 new diagnosed patients worldwide per year [1]. GBM is typically associated with bad prognosis for survival, with a fatal outcome within 12 to 18 months after diagnosis [2–5]. Combination therapies with temozolomide and radiation are now used worldwide; however, they did not lead to a significant improvement of the life expectancy of the patients, thus the need for more effective treatments is still and urgently persisting [6].

Over the past decade, evidence is growing on the heterogeneous nature of brain tumors [7–9]. Among their various differentiated cells, these tumors contain a core of stem-like cells, which in the case of GBM are known as glioblastoma stem cells (GSCs). These stem-like cells are usually not completely removed by surgery, are resistant to chemotherapy and radiotherapy, and thus responsible for aggressive tumor recurrence. Therefore, new approaches for early GBM diagnosis and treatment aim at the selective targeting of the GSCs, for which novel GSC-specific biomarkers need to be identified [10].

Unlike small-molecule therapeutics used in the treatment of cancers, monoclonal antibodies that recognize specific targets of the pathogenic pathways provide a far greater specificity and offer an alternative with many advantages. Indeed, antibody therapies directed against several types of molecules that have a role in carcinogenesis have demonstrated significant success in the past decade [11, 12]. Blood-brain barrier permeability within different areas of the same tumor is variable since the integrity of the blood-brain barrier is highly heterogeneous within tumor tissue [13]. However, when targeting molecules to the brain, only 0.02% to 0.10% of conventional immunoglobulins in serum can penetrate into the brain parenchyma [14]. Brain penetration of high affinity antibodies against novel biomarkers thus appears to be seriously limited by the blood–brain barrier. However, it has been demonstrated that engineered heavy-chain variable domains (VHHs) with a basic isoelectric point can readily transmigrate across the blood–brain barrier *in vivo* after peripheral injection, without the need for any invasive procedure to weaken the blood–brain barrier [15]. These VHHs, also known as nanobodies, are single-domain antibody fragments derived from camelid heavy-chain-only antibodies [16–20]. Using various approaches for selection, nanobody libraries are used to retrieve a desired nanobody with high stability, affinity and specificity towards its cognate antigen [21, 22]. Nanobodies have significant advantages compared to standard antibodies that make them useful candidates as biopharmaceuticals and imaging tools. With their small size, they can easily reach hidden or cryptic targets; they also rapidly bind tumor antigens, specifically *in vivo*, whereas excess nanobodies are eliminated rapidly from the blood stream *via* the kidneys [23, 24]. Besides their use as versatile bio-imaging tools in living cells, nanobodies have been used as valuable *in vivo* detection probes for cancers, infectious diseases, atherosclerotic lesions, inflammatory responses, and many other diseases, in both preclinical and clinical environments [25].

In our previous study [26], a llama was immunized with whole human GBM cells enriched in GBM stem cells. Messenger RNA was isolated from the llama lymphocytes and used to construct an immune phage-displayed VHH library. Immunoaffinity enrichment was performed on protein isolates from GBM tissue, *versus* normal brain tissue.

In the present study, enrichment of the phage-display nanobody library was performed using immunoaffinity selection (bio-panning) with the NCH421K and NCH644 GBM stem cell lines to expand the pool of cancer stem cell specific nanobodies. The mitochondrial translation elongation factor (TUFM) was identified by mass spectrometry (MS) as the target antigen of a GSC specific nanobody and validated by western blot and RT-qPCR. The anti-TUFM nanobody specificity towards its antigen was confirmed by *in silico* three-dimensional (3D) modelling and immunocytochemistry. Furthermore, this anti-TUFM nanobody (referred to as Nb206) exerts a negative effect on GBM stem cell growth, and we propose its future application as a lead for *in vitro* immunoimaging and GBM stem cell targeting.

## RESULTS

### Immunoaffinity selection and antigen identification

**Figure 1:**
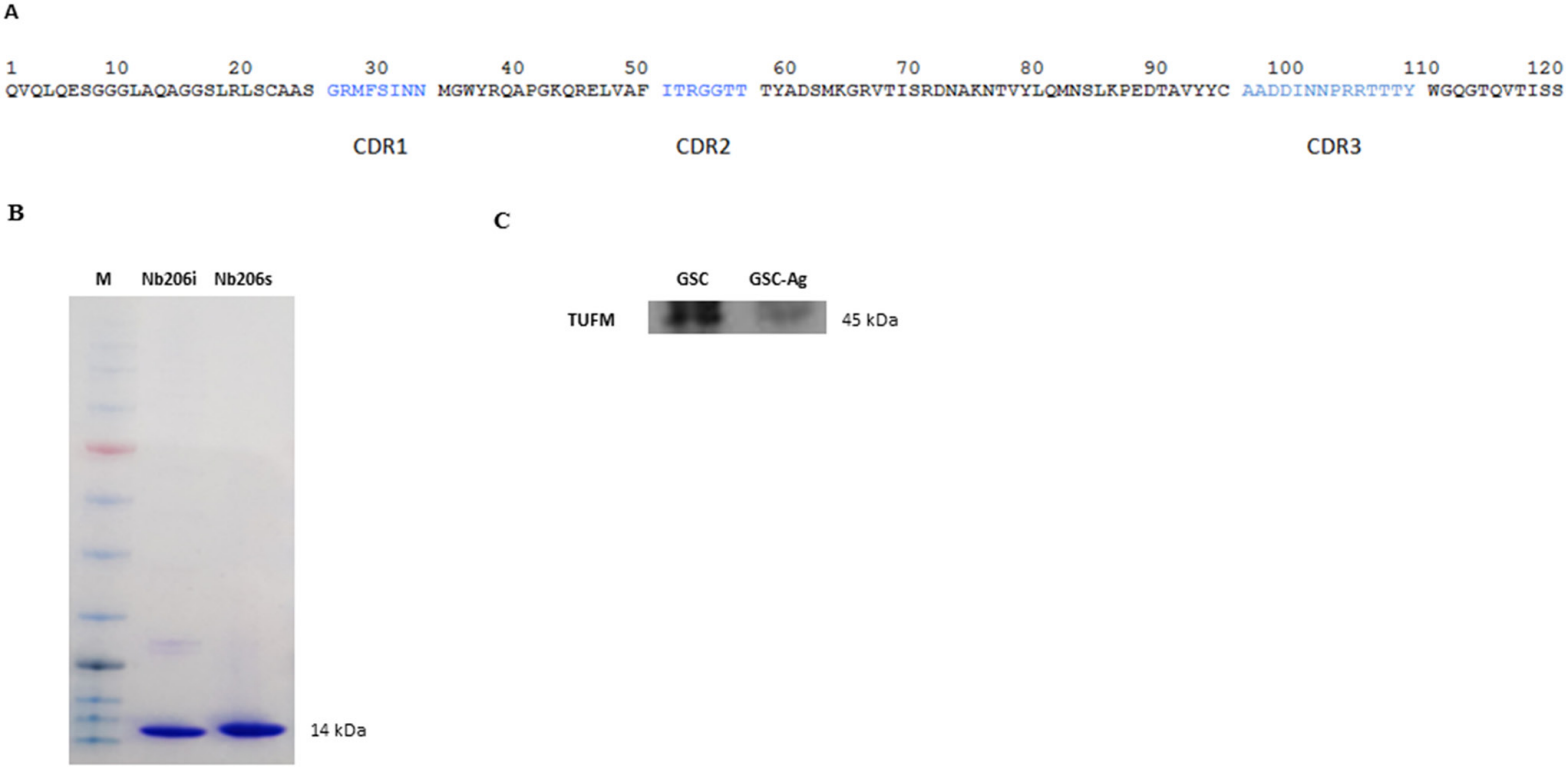
**(A)** Nanobody amino-acid structure. In the H1 loop (CDR1), the Arg27 is often observed at this position in llama VHH, but is usually substituted by Tyr in dromedary VHH. This suggests that a *VHH* germline gene occurs in llama with the sequence GRTFSS. In framework region 2, the amino-acid sequence KQREL is often observed and is a hallmark motif for soluble, stable nanobodies. The amino-acid sequence of the H3 loop (CDR3) is given in alphabetical order. **(B)** SDS-PAGE gel analysis of a Nb206. M, Blue Star Prestained Protein Marker; Nb206i, sample of Nb206 after immobilized metal affinity chromatography; Nb206s, sample of Nb206 purified with size exclusion chromatography. Size of the Nb206 corresponds to a 14kDa. **(C)** Western blot of glioblastoma stem cell lysate (GSC) and GSC lysate used for specific binding of TUFM on Ni beads-Nb206 (GSC-Ag).

The phage-displayed nanobody library comprised 10^8^ individual transformants of which 80% had an insert in their pHEN4 vector that corresponded to the size of a nanobody gene. For immunoaffinity enrichment, whole GSCs were used (i.e., NCH644 and NCH421K cells). Three rounds of bio-panning were performed, and 480 single bacterial clones were screened. Eight different clones (nanobodies) that showed at least 1.9-fold higher enzyme-linked immunosorbent assay (ELISA) signal in GSC than in normal tissue lysates (Supplementary Table 1) were sequenced to determine amino-acid sequences. From these eight nanobody sequences only one nanobody, defined as Nb206, revealed the starting (QVQL) and the ending (TISS) amino-acid sequence of a whole VHH (Figure 1A). With recloning into the pHEN6 vector, a polyhistidine-tag was introduced to facilitate its purification by immobilized metal affinity chromatography. After subsequent purification by size-exclusion chromatography, Nb206 was applied to 4%-12% NuPAGE Bis-Tris mini gel (Invitrogen) to confirm its purity and the expected molecular weight of 14 kDa (Figure 1B). We immobilized purified Nb206 to Ni-TED resin and captured its antigen from the cell lysate (Figure 1C). The MS analysis (Supplementary Figure 1) identified the corresponding antigen of Nb206 as the mitochondrial translation elongation factor TUFM, which has also been referred to as EF-TU.

### Validation and quantification of TUFM protein

**Figure 2:**
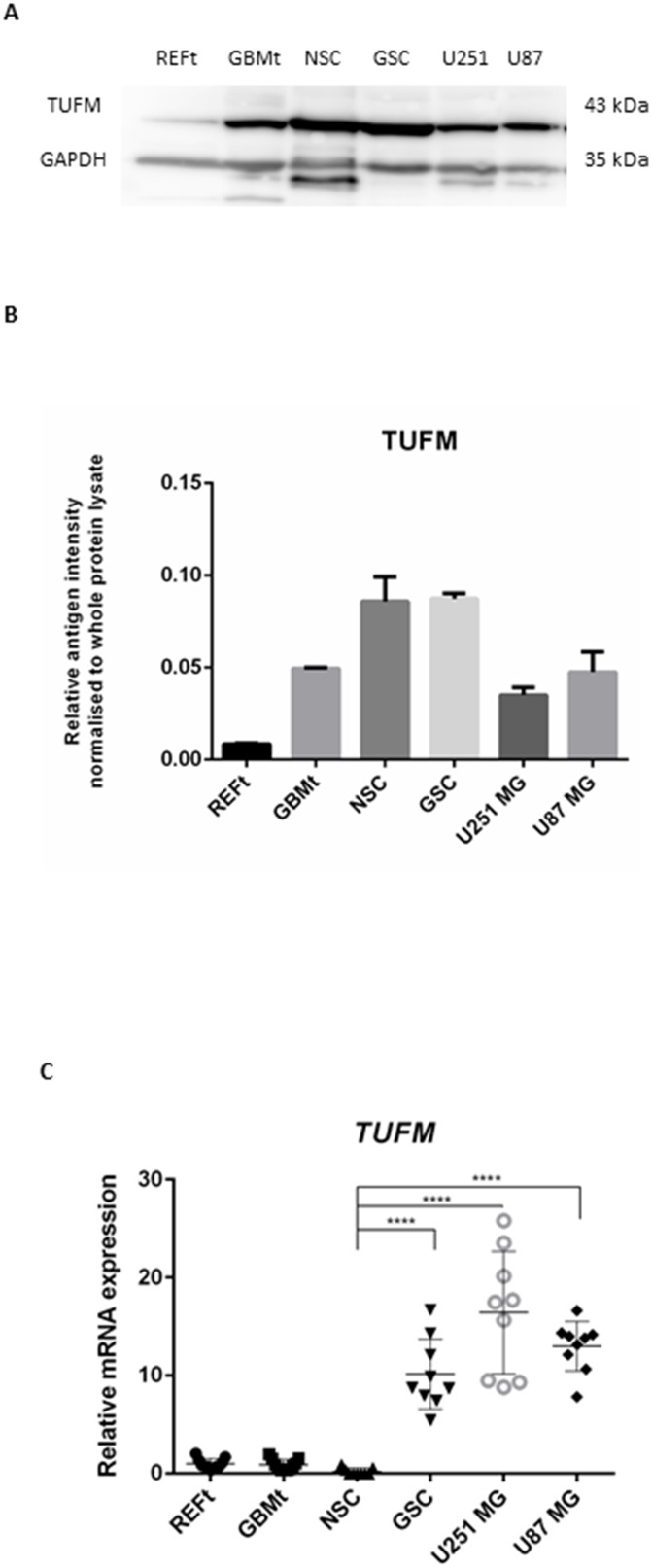
Protein and mRNA expression of TUFM **(A)** Representative western blot of normal brain tissue (REFt), glioblastoma tissue (GBMt), glioblastoma stem cells (GSC), neural stem cells (NSC) and two commercial glioblastoma cell lines, U251MG and U87MG. GAPDH was used as the loading control. **(B)** Quantification of western blotting for TUFM expression bands as shown in Figure 2A, for REFt, GBMt, GSC, NSC, U251MG and U87MG. Relative band intensities were calculated as the ratios between the antigen band intensities for TUFM and those of the whole protein. **(C)** *TUFM* relative mRNA expression in tissue samples was calculated as the ratio between GBMt and REFt mRNA expression. Relative mRNA expressions in cell lines were calculated as the ratios between GBM cell lines and NSC expression. ^*^ p <0.05, ^**^ p < 0.01, ^***^p < 0.001, ^****^ p < 0.0001.

Western blotting was employed to validate the expression of TUFM protein in normal brain tissue, GBM tissue, neural stem cells (NSCs), GSCs, and U251MG and U87MG GBM glioblastoma cell lines (Figure 2 and Supplementary Figure 2). TUFM bands were quantified relative to the whole protein lysates, as the ratio between the TUFM band intensity and the whole protein lysate band intensity (both measured in arbitrary units [AU]) (Figure 2). Statistical p values of western blot analysis are shown in Table 1.

**Table 1:** Statistical p value of western blot analysis for TUFM

Sample comparison		P value
REFt *vs.* GBMt	^****^	< 0,0001
REFt *vs.* NSC	^****^	< 0,0001
REFt *vs.* GSC	^****^	< 0,0001
REFt *vs.* U251MG	^***^	0,0009
REFt *vs.* U87MG	^****^	< 0,0001
GBMt *vs.* NSC	^****^	< 0,0001
GBMt *vs.* GSC	^****^	< 0,0001
GBMt *vs.* U251MG	ns	0,1042
GBMt *vs.* U87MG	ns	0,9985
NSC *vs.* GSC	ns	0,9997
NSC *vs.* U251MG	^****^	< 0,0001
NSC *vs.* U87MG	^****^	< 0,0001
GSC *vs.* U251MG	^****^	< 0,0001
GSC *vs.* U87MG	^****^	< 0,0001
U251MG *vs.* U87MG	ns	0,2087

The relative band intensity for TUFM was 10 % higher in GSCs (0.0862) compared to NSCs (0.0778) (p value ns). Quantification of TUFM expression in the NSCs also revealed a 2.5-fold and 2.0-fold increase compared to those for the two GBM cell lines, U251MG (0.0316) (p < 0.0001) and U87MG (0.0383) (p < 0.0001) cells, respectively. As concerning the GBM tissue (0.0496) the relative band intensity for TUFM was 5.6-fold higher than in the reference tissue (0.0088) (p < 0.0001) (Figure 2B).

### Quantification of *TUFM* mRNA expression in GBM tissue

The mRNA was successfully isolated from all of the GBM and normal samples, and showed a mean concentration of 910 ng/μL and a 260 nm to 280 nm absorption ratio (A_260_/A_280_) from 1.81 to 2.02. The mean concentration of mRNA isolated from NSCs, GSCs and the two glioblastoma cell lines, U87MG and U251MG, was 1835 ng/μL, with an A_260_/A_280_ ratio from 1.92 to 1.99.

As the best gene combination for reference genes, NormFinder selected *RPL13A* and *CYC1*, with a stability value of 0.028. The PCR primer sequences are presented in Table 2. For the reference genes, the primer efficiency was from 1.82 for *CYC1* to 2.26 for *HPRT1*.

**Table 2:** Primers for the candidate reference and target genes

Gene group	Gene name	Primer sequence (5’→3’)
Reference	*TBP*	F: CAG CAT CAC TGT TTC TTG GCG T
		R: AGA TAG GGA TTC CGG GAG TCA T
	*HPRT1*	F: CAG CCC TGG CGT CGT GAT TAG T
		R: CCA GCA GGT CAG CAA AGA AT
	*RPL13A*	F: CCT GGA GGA GAA GAG GAA AGA GA
		R: TTG AGG ACC TCT GTG TAT TTG TCA A
	*GAPDH*	F: TCG CCA GCC GAG CCA CAT C
		R: CGT TCT CAG CCT TGA CGG TGC
	*CYC1*	F: GAG GTG GAG GTT CAA GAC GG
		R: TAG CTC GCA CGA TGT AGC TG
Target	*TUFM*	F: AAA GAA GGG AGA CGA GTG TGA
		R: TGT GGA ACA TCT CAA TGC CTG

F, forward; R, reverse.

Intergroup comparisons for *TUFM* mRNA expression did not show any major differences between the glioblastoma tissue (GBMt) and normal brain tissue (REFt) samples (p = 0.5934). However, *TUFM* mRNA expression was significantly greater for GSCs (p < 0.0001), U251MG (p < 0.0001) and U87MG (p < 0.0001) GBM cell lines compared to NSCs (Figure 2C).

### Cytotoxicity of Nb206 in different cell lines

**Figure 3:**
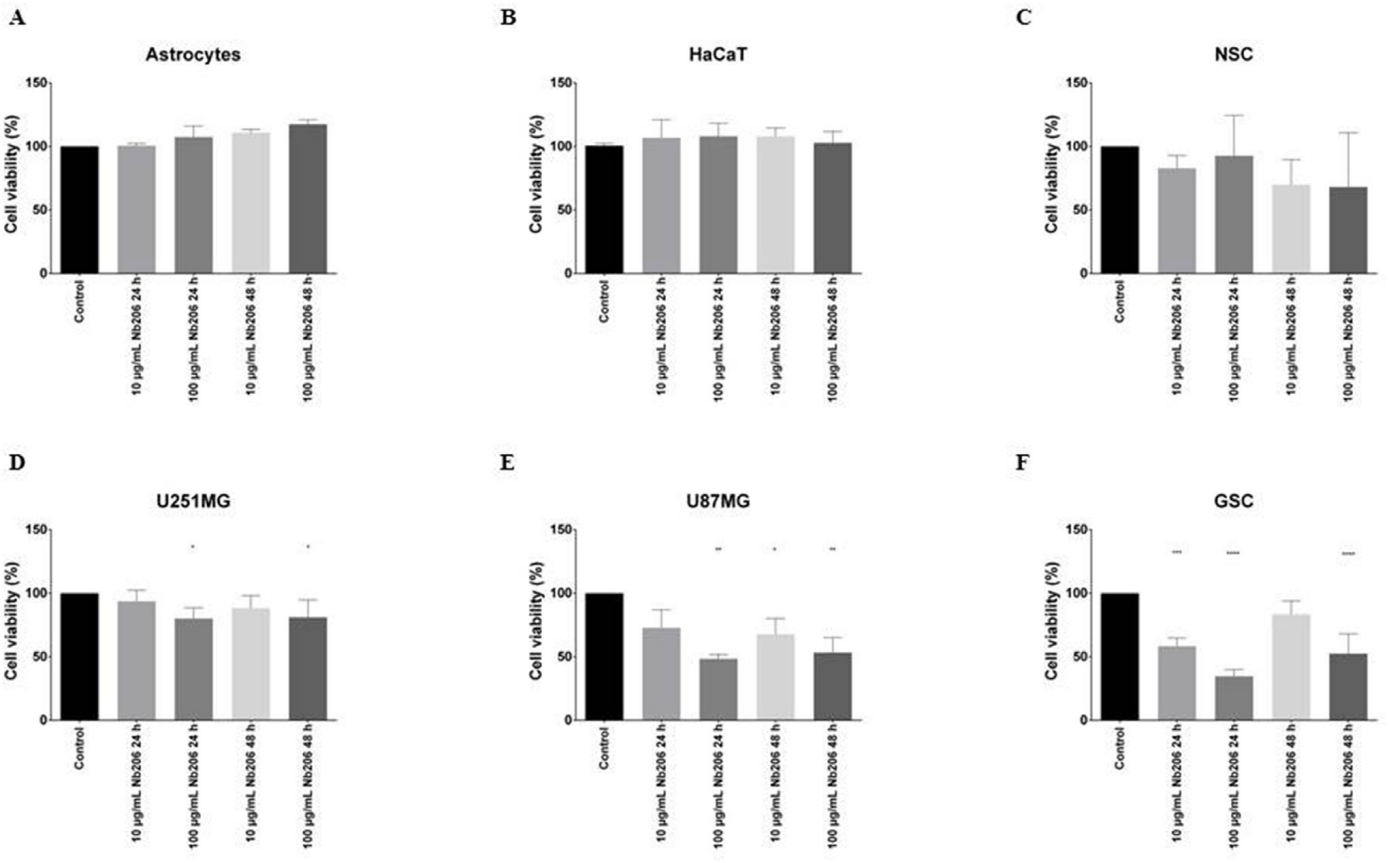
Cytotoxic effects of Nb206 (anti-TUFM nanobody) on the different cell lines, as indicated Data are means ±SD. ^*^ p < 0.05, ^**^ p < 0.01, ^***^ p < 0.001, ^****^ p < 0.0001, *versus* respective control.

To examine whether the Nb206 can suppress cell growth, cytotoxicity was measured, according to the WST-1 metabolic assay reagent, on the GBM cell lines and using the following control cells: NSCs, astrocytes, and a spontaneously immortalized human keratinocyte cell line (HaCaT cells). Potential effects were determined at both 24 h and 48 h after the addition of 10 μg/mL and 100 μg/mL Nb206. The data shown in Figure 3 confirm that Nb206 significantly inhibited in a dose-dependent manner the proliferation of GSCs and the U87MG GBM cell line in particular, and to a lesser extent the U251MG cells (Figure 3D-3F). Thus, the mean cell viabilities after 24 h with 100 μg/mL Nb206 were significantly reduced, especially for GSCs (34.7%; p <0.0001) but also of U87MG (48.5%; p = 0.0036) and U251MG (80.2%; p = 0.0285) cells. Significance was still seen at 100 μg/mL Nb206 after 48 h, as 52.5% (p <0.0001), 53.3% (p = 0.0015) and 81.1% (p = 0.0395) viability, respectively.

For NSCs, astrocytes and HaCaT cells, there was no significant reduction in cell growth with up to 100 μg/mL of Nb206 over 48 h (Figure 3A, 3B, 3C).

### Apoptosis and necrosis of glioblastoma cell lines and glioblastoma stem cells

**Figure 4:**
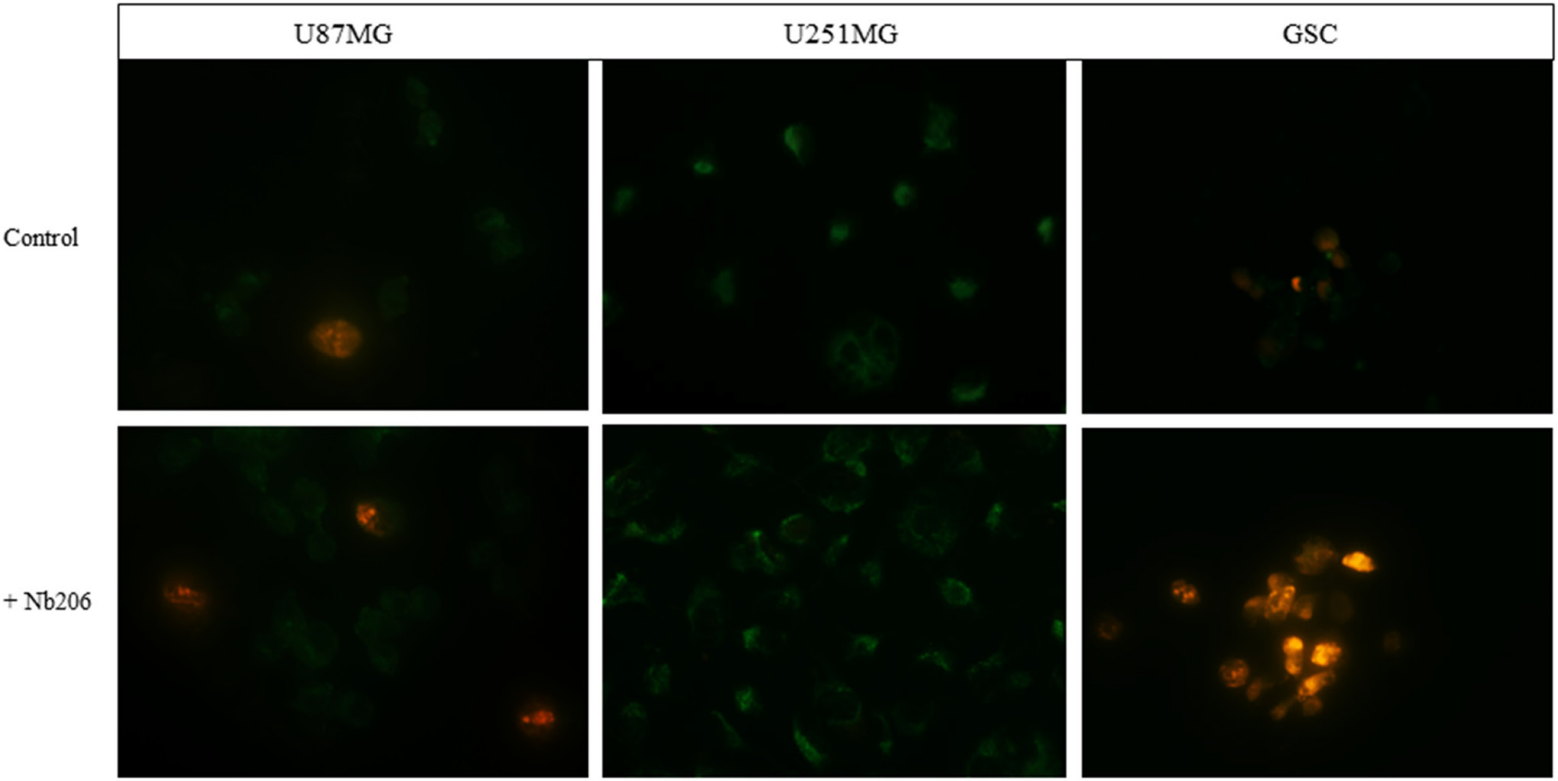
Apoptosis/necrosis test on glioblastoma cell lines U87MG and U251MG and glioblastoma stem cells Annexin V: FITC conjugated (green), apoptotic cells, Propidium Iodide (red), necrotic cells.

Apoptosis and necrosis were observed in 2 different glioblastoma cell lines U251MG and U87MG and in GSC (Figure 4) using Annexin V-FITC/PI. In comparison with suitable controls, upon treatment with Nb206, GSC and U87MG cells are subjected to progressive apoptosis (green) and necrosis (red). Remarkably, necrosis was not observed in the U251MG cell line, while in comparison apoptosis is progressing in the control U251MG cells.

### Visualization of three-dimensional interaction between Nb206 from Lama glama and its target, human mitochondrial elongation factor EF-TU

**Figure 5:**
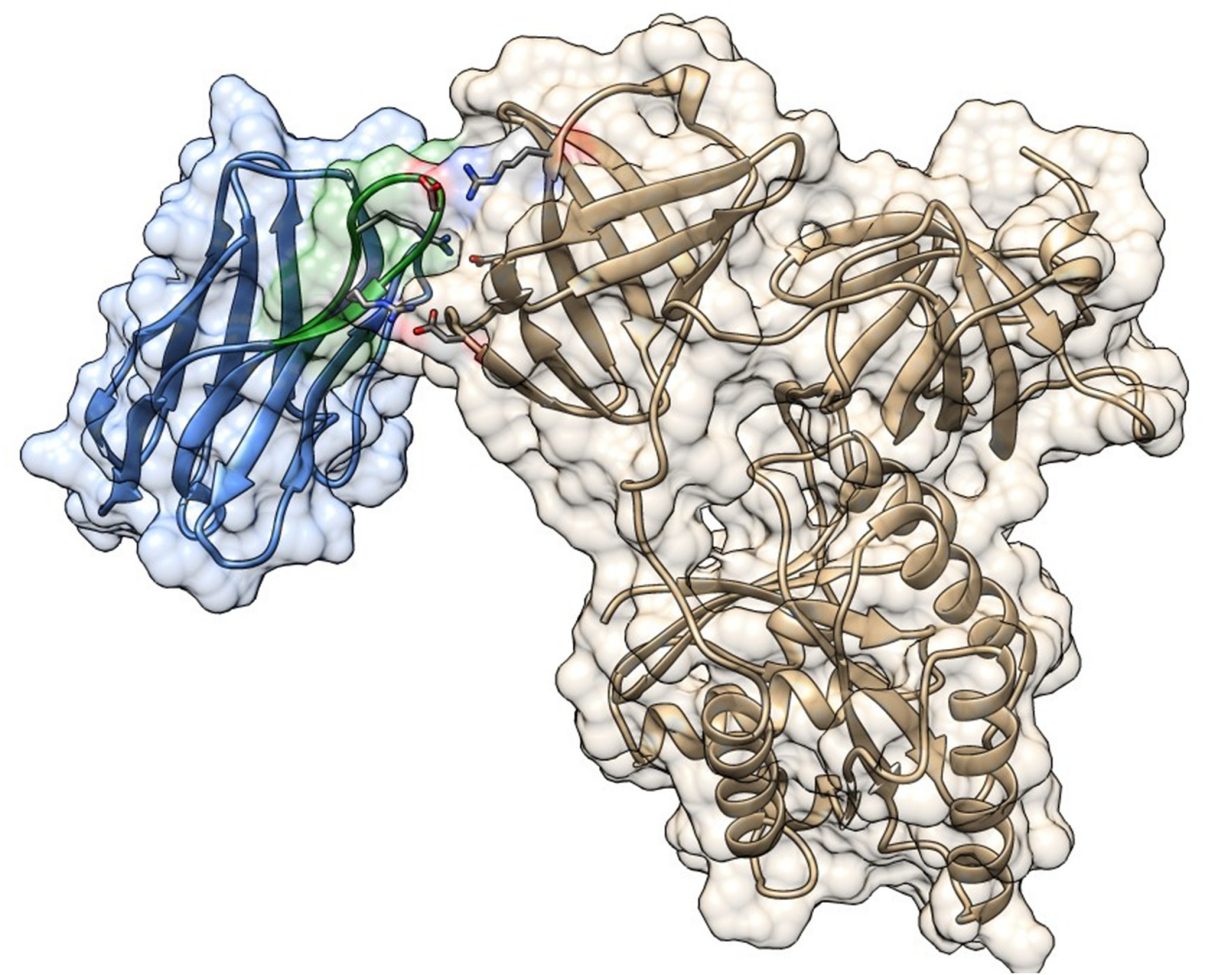
Three-dimensional model for the complex between Nb206 (metal-blue with CDR3 in green) from *Lama glama* and its target human mitochondrial elongation factor (EF-TU/ TUFM; gold) The final frame after 400 ns of molecular dynamics simulation is shown in ribbon representation embedded in surface contours with the Nb-CDR3 Arg99, Asp103 and Arg106 in sticks and in CPK coloring and the Glu253, Asp260 and Arg 273 of TUFM in sticks and CPK coloring. Molecular graphics were performed with UCSF Chimera [50].

**Figure 6:**
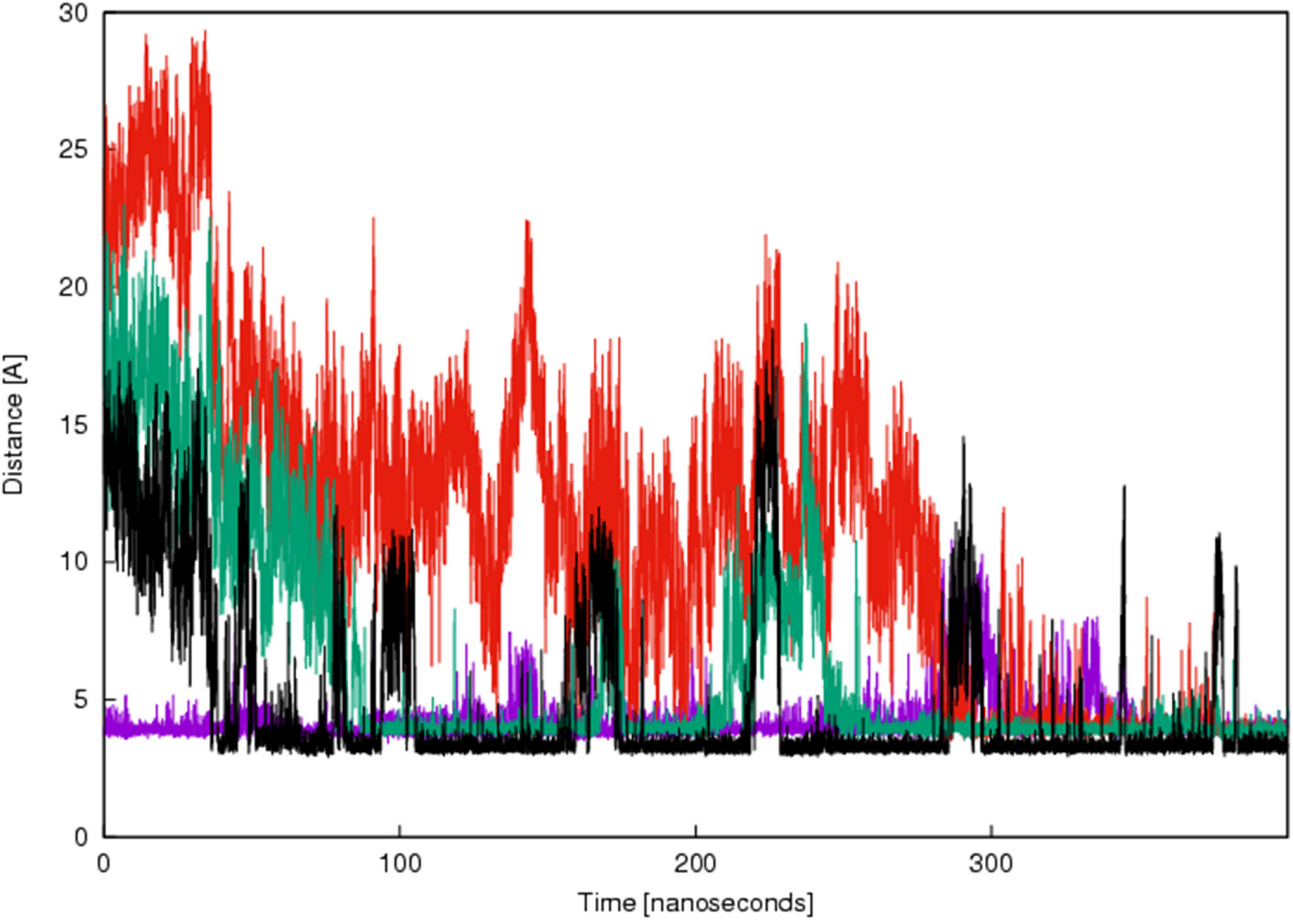
Time-resolved changes in the distances between the selected opposite charged amino acids in Nb206 and EF-TU/TUFM Violet, R100 and E253; red, R107 and D260; green, D104 and R273; black, D98 and K257. X axis shows time from the equilibration of the complex formed between Nb206 and EF-TU. Y axis shows distance between the selected opposite charged amino acids in Nb206 and EF-TU in angstrom (Å).

**Figure 7:**
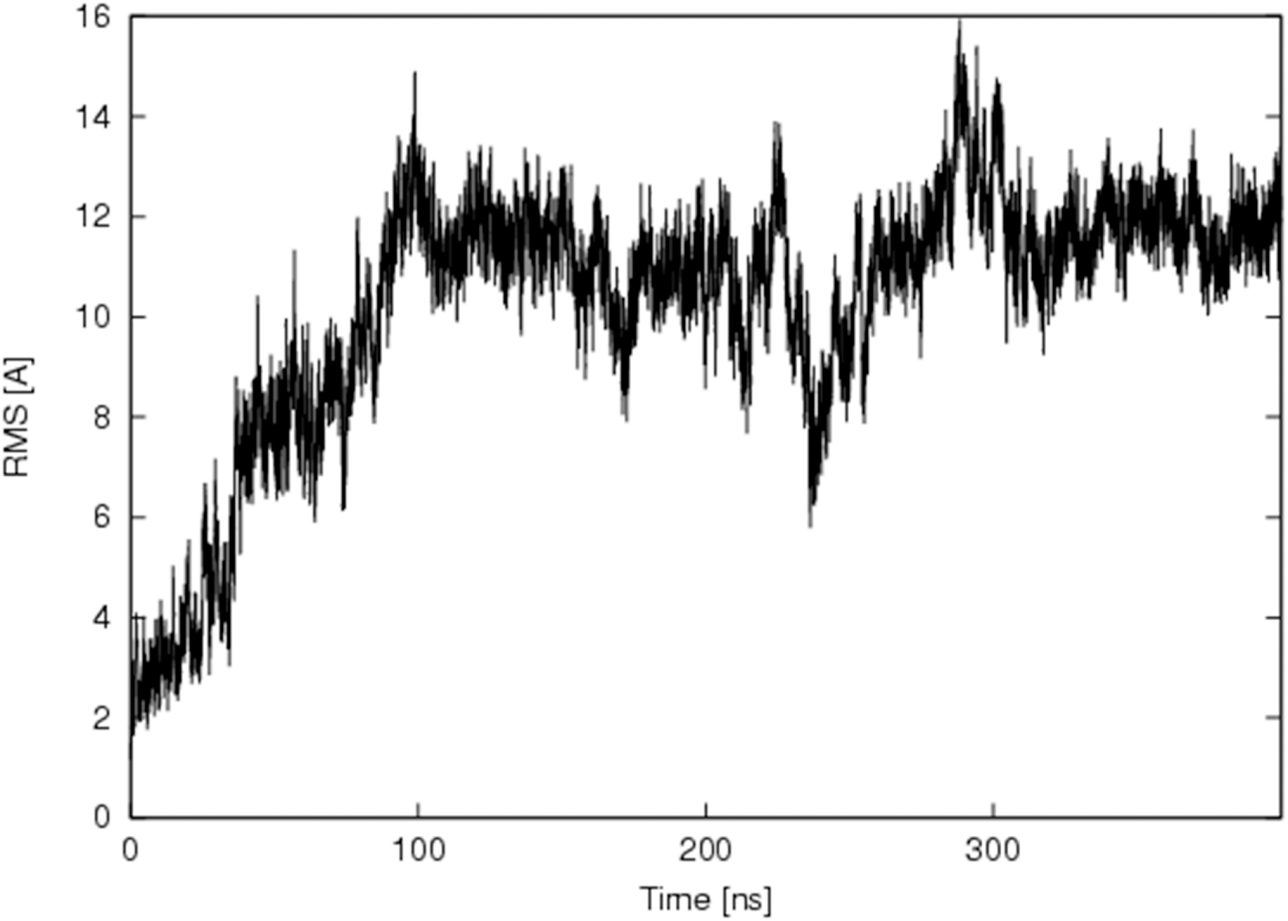
Time resolved changes in root mean square for the Nb206–human elongation factor complex over 400 ns of the molecular dynamics simulation run X axis shows time from the equilibration of the complex formed between Nb206 and EF-TU. Y axis shows root mean square in angstrom (Å).

The 3D structure of the interaction between Nb206 and human EF-TU (TUFM) has not been solved. We therefore searched for a suitable homology template to build upon. High sequence identity (76%) was seen between Nb206 and a camelid nanobody from the solved protein-protein complex for PDB entry 4WGV, with only one single amino-acid deletion [27]. Therefore, the ALIGN and BLDPIR commands in the WHATIF modelling suite [28] were used for homology building of Nb206 with this template. Figure 5 shows the final modelled complex of the camelid Nb206 (metal-blue, CDR3 region in green) and human EF-TU (gold) after 400 ns of a molecular dynamics run. From Figures 5, 6 and 7, it can be seen that Nb206 and human EF-TU remain in close contact throughout the entire analysis period. Indeed, Figure 7 indicates a significant increase in the root mean square during the simulation (app. 15 Å), although this is a consequence of the refined accommodation at the protein–protein surface, with the subsequent change in the main angle of the interaction. Moreover, it is also clear from Figure 7 that at approximately 100 ns the root mean square was equilibrated, with indications of periodic fluctuations around a mean conformation. The same is true for the distances between certain of the opposite charged residues in the Nb206 and the human EF-TU protein (Figure 6). On the other hand, a second simulation run where the C-terminal domain of Nb206 was positioned to interact with the target EF-TU resulted in complete dissociation of these two proteins after only 200 ns, with no possibility of any further interaction (data not shown).

### Immunocytochemistry for Nb206

**Figure 8:**
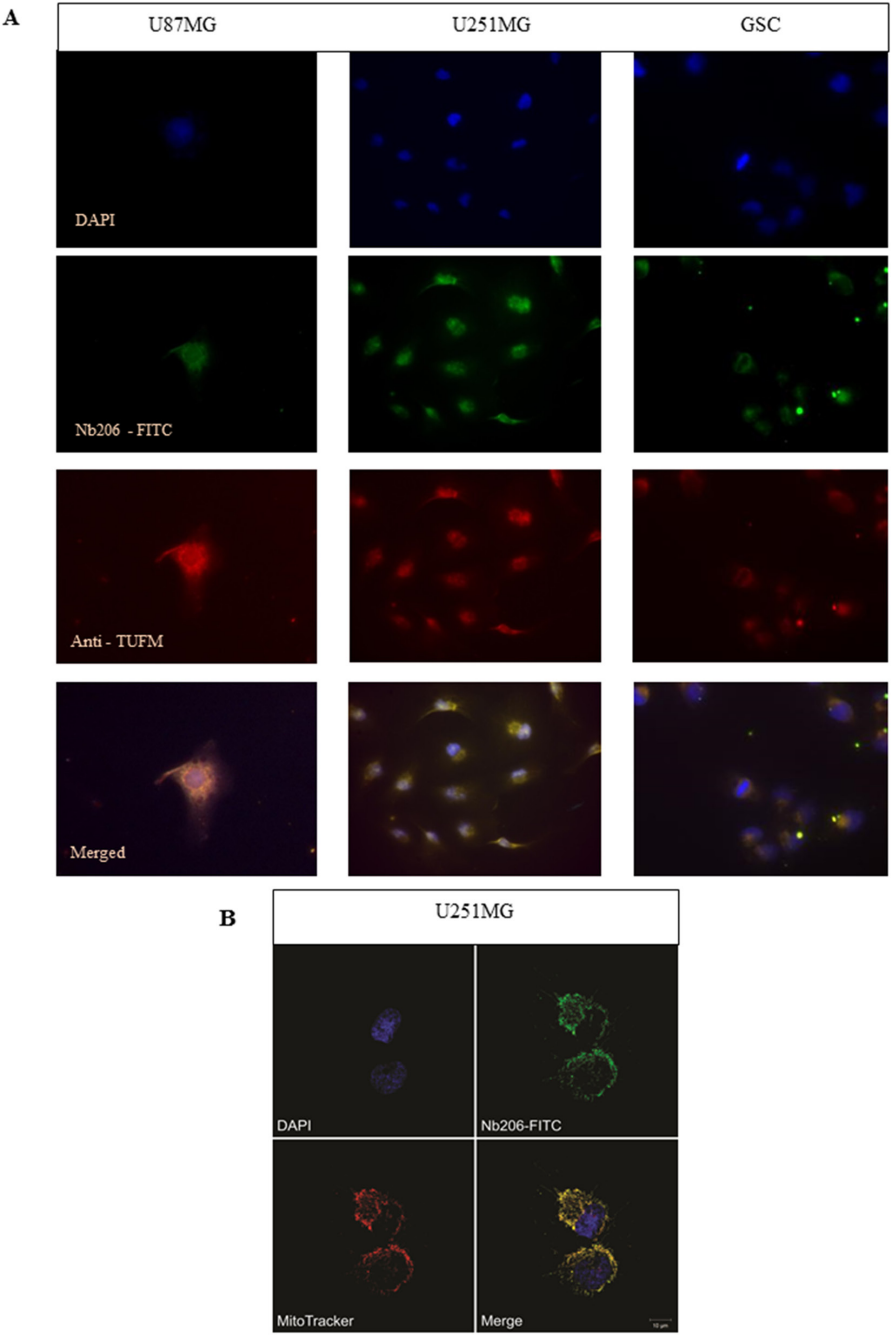
**(A)** Immunocytochemistry with the U87MG (left) and U251MG (middle) glioblastoma cell lines and the GSCs (right), as examined using fluorescence microscopy. DAPI, blue, nuclei; Nb206-FITC conjugated, green, anti-TUFM; commercial monoclonal anti-TUFM antibody, red; merged signals (bottom row). **(B)** Immunocytochemistry with U251MG glioblastoma cell line examined with confocal microscopy. Hoechst 33342, blue, nuclei; Nb206 - FITC conjugated, green, anti-TUFM; MitoTracker Orange dye, red, mitochondrial filaments and clusters.

To further confirm specificity of binding of Nb206 to the TUFM antigen, *in vitro* immunocytochemistry was performed on whole GBM cells (Figure 8A). GSCs, U251MG and U87MG were grown on glass slides, immobilized and stained with either Nb206 conjugated with FITC (Figure 8A, green) or with a commercial anti-TUFM antibody combined with secondary antibody anti-mouse IgG (H+L) F(ab`)2 CF640R (Figure 8A, red). The cell nuclei were stained with DAPI (Figure 8A, blue). Nearly 100% of the cells stained positively for both Nb206 and the anti-TUFM antibody. Indeed, the merged images for Nb206 and the anti-TUFM antibody (Figure 8, bottom) illustrate the overlap in signal mainly around the cell nuclei, which is consistent with the proposed localization of TUFM (http://www.proteinatlas.org/ENSG00000178952-TUFM/cell). To confirm colocalization of Nb206-conjugated with FITC (Figure 8B, green) with mitochondrial filaments, we stained mitochondria using MitoTracker Orange dye (Figure 8B, red) and observed signals under confocal microscope. The cell nuclei were stained with Hoechst 33342 (Figure 8B, blue). The signals for Nb206 and mitochondrial network overlapped and are seen in yellow.

## DISCUSSION

In the treatment of patients with GBM, new promising approaches are urgently needed that should be focused on the targeting of GSCs. The aim of our current study is to identify and determine the functions of novel GSC target proteins, while searching for proteins with reduced or even without expression in NSCs [10, 29]. As described previously [26], a llama was immunized with the whole fraction of GBM stem-like cells to raise an immune response towards the cell-surface proteins. After immunizing a llama, its lymphocyte mRNA was used to synthesize a cDNA ‘immune’ library of nanobodies, and then to construct the corresponding phage-displayed nanobody library. Through bio-panning of this library with GSCs, we enriched the nanobodies that targeted proteins that are overexpressed in GSCs. Remarkably, from a number of nanobodies that had good ELISA signals, only one, Nb206, showed the starting and ending amino-acid sequences of a nanobody. The consequent proteomic analysis identified the corresponding antigen of Nb206 as TUFM (or EF-TU).

We reasoned that this intracellular mitochondrial protein (i.e. TUFM) was identified because of lysis of the glioma cells that would have occurred during the immunization and panning procedures (which was performed with whole GSCs), and also because the screening was performed on GSC protein lysates. TUFM was recognized among seven possible glioma tumor-class predictive biomarker candidates in our previous study [30].

*TUFM* is a nuclear gene that encodes a protein that is synthesized in the cytoplasm and transported into the mitochondria, where it regulates expression of the mitochondrial genome by controlling the translation of mtDNA-encoded proteins. Mutations in the *TUFM* gene have been associated with combined oxidative phosphorylation deficiency, which results in lactic acidosis and fatal encephalopathy (https://targetexplorer.ingenuity.com/gene/EG/7284/pathways). *TUFM* knock-down induces epithelial-to-mesenchymal transition (EMT), reduced mitochondrial respiratory chain activity, increased glycolytic function, and production of reactive oxygen species. Decreased expression of mtDNA induces mitochondrial dysfunction, with cell stresses such as ATP deficiency and reactive oxygen species production, which trigger AMPK activation and the subsequent regulation of nuclear gene expression and EMT [31]. Mani *et al.* (2008) suggested that cells that undergo EMT can gain stem cell like properties, thus giving rise to cancer stem cells [32]. Chaffer *et al.* (2011) reported the involvement of EMT and its reverse, mesenchymal-to-epithelial transition, in the initiation of metastatic processes that can enable circulating tumor cells to form micro-metastases and to express clonal outgrow at metastatic sites [33]. In 2015, Seyfried [34] developed an interesting view on the mitochondrial origin of cancers, on the basis that cancers originate from damage to the mitochondria rather than from damage to the cell genome, and that the genomic damage in tumor cells follows, rather than precedes, the disturbances in cell respiration. Indeed, in the case of TUFM, a direct association between its down-regulation and induction of EMT and carcinogenesis was shown for lung cancer [35], and its down-regulation as an indicator of an advanced stage of disease was shown for gastric cancer [36]. On the other side, TUFM overexpression was reported negative prognostic factor for colorectal cancer [37] and human pancreatic adenocarcinoma [38]. Here, we present evidence for TUFM overexpression in GBM and glioblastoma stem cells.

Validation with western blotting showed that TUFM was detected at the protein level in all GBM cell lines and GBM tissue, and also in NSCs. The very aggressive and proliferative GSCs that were used in the present study (i.e., a coculture of NCH644 and NCH421K cells) revealed the highest intensity of TUFM protein expression, compared to the mature U251MG and U87MG GBM cell lines. At the mRNA level, we confirmed that *TUFM* expression in GSCs and U251MG cells was significantly elevated compared to NSCs. At the protein level, TUFM was also overexpressed in GBM tissue compared to normal brain reference tissue, although there was no correlation with *TUFM* expression at the mRNA level. The protein stability might be increased due to different reasons like post-translational modifications, or protein accumulation over time, while mRNA turnover is a particularly rapid process [39].

The high protein content of TUFM in NSCs and GSCs suggests their potential as candidate biomarker for cell stemness, as well as being specific GBM biomarker due to its relative overexpression in all of the GBM-related cell types, in contrast to the normal brain tissue.

We also compared the cytotoxic effects of Nb206 on GBM cell lines. Nb206 had no influences on astrocyte as well as on HaCaT cell growth. After 24 h and 48 h treatment Nb206 showed little or no inhibitory dose-independent effect on NSC growth. Instead, Nb206 showed its greatest cytotoxic effects on GSCs and the U87MG GBM cell line. After 24 h of treatment with 100 μg/mL Nb206, cell survival was reduced to 34.7% for the GSCs and 48.5% for the U87MG cells, which after 48 h was seen slightly higher, as 52.5% and 53.4% survival, respectively, which again might be attributed to some proteolytic degradation or export of Nb206 over time.

Nanobodies are stable molecules that often bind to catalytic sites, and thus they can also be exploited as *in vivo* immunomodulators (i.e. intrabodies) that can interfere with protein conformation, localization and/or function [40]. To determine the utility of Nb206 in immunocytochemistry, we stained three different GBM cell lines with a commercial anti-TUFM antibody and also with Nb206 conjugated with FITC. Indeed, after overlaying the images, these immunofluorescence signals superimpose. Colocalization of Nb206 conjugated with FITC with mitochondrial filaments examined with confocal microscopy confirmed these results. Therefore, we propose that Nb206 can be used as a tool for the detection of TUFM in immunocytochemistry.

To comprehensively interpret the specificity or binding affinity of Nb206 towards its antigen, TUFM, relatively long molecular dynamics simulations were performed that suggested a stable interaction of Nb206 from *Lama glama* with human EF-TU. The control simulation with modified Nb206 orientation resulted in its complete dissociation after a shorter run, thus enhancing the significance of the stable complex.

In conclusion, Nb206 showed strong cytotoxic effects on GSCs and U87MG cells, and mild cytotoxic effects on U251MG cells, all of which are specific GBM cell lines. These effects were not seen for the ‘normal’ brain cell lines, such as astrocytes and NSCs, and for the more distant HaCaT cell line. Furthermore, strong antigen binding affinity of Nb206 was supported by *in silico* 3D modelling, which showed strong interactions between the crucial amino acids, as Arg 103 in the Nb206 complementarity determining region-3 binding domain, and Asp 260 of TUFM. Due to significant overexpression of its antigen, TUFM, in GBM tissue and GSCs as compared to normal brain tissue, we recommend Nb206 as suitable candidate for *in vitro* immunoimaging. Furthermore, we recommend its use in the GBM targeting experiments, due to its prolonged cytotoxic effects in all GBM-associated cell lines that can last even after 48 h of treatment.

## MATERIALS AND METHODS

### Cell lines

The U251MG and U87MG GBM cell lines (ATCC) were cultured in high glucose (4.5 g/L D-glucose) Dulbeccoʼs modified Eagleʼs medium (DMEM; Invitrogen, Life Technologies), supplemented with 10% fetal bovine serum, 2 mM L-glutamine, and antibiotic antimycotic solution (Cat. No. 15240062) (all from Invitrogen, Life Technologies). GSCs (coculture of NCH644 (https://clsgmbh.de/pdf/nch644.pdf) and NCH421K (https://www.clsgmbh.de/pdf/nch421k.pdf) cells, both from CLS) were grown as spheroid suspensions in complete Neurobasal Medium supplemented with GlutaMAX, antibiotic antimycotic, B-27, 20 ng/mL bFGF, 20 ng/mL EGF (all from Invitrogen, Life Technologies) and 1 U/mL heparin (Sigma-Aldrich). The human NSCs, h9 derived (Gibco; https://assets.thermofisher.com/TFS-Assets/LSG/manuals/GIBCO_hNSC_man.pdf) were cultured in KnockOutTM DMEM/F-12 Basal Medium supplemented with GlutaMAX, antibiotic antimycotic, 1 mL StemPro neural supplement, 20 ng/mL EGF, 20 ng/mL bFGF (all from Invitrogen) and 1 U/mL heparin (Sigma-Aldrich). Once the GSC and human NSC spheroids reached approximately 200 nm in diameter they were dissociated with vigorous pipetting. Human astrocytes were cultured in Astrocyte Medium supplemented with penicillin/ streptomycin and astrocyte supplement (all from ScienCell), with the attachment surface pre-treated with poly-D-lysine. HaCaT cells are a spontaneously immortalised human keratinocyte cell line (ATCC) and they were cultured in DMEM with 10% fetal calf serum supplemented with antibiotic antimycotic solution. All of the cell lines were grown in an incubator at 37°C and 5% CO_2_.

### Protein samples

For ELISA screening and western blot validation, proteins were extracted from all samples (GBM tissues, GSC, U251 MG and U87MG GBM cell lines, NSC and non-tumor brain tissues) using ProteoExtract^®^ Transmembrane Protein Extraction Kit (Novagen^®^) and ProteoExtract^®^ Native Membrane Protein Extraction Kit (Calbiochem^®^). *Post-mortem* brain samples were provided by the Institute of Pathology, University Clinical Centre in Ljubljana, Slovenia. Concentration of proteins was measured with BCA assay.

### Immunoaffinity enrichment (bio-panning)

For this study, we used a phage-display VHH library that was constructed previously. Phage enrichment was performed on whole cells of GBM stem cell lines, as NCH644 and NCH421K cells, provided by Christel Herold-Mende (University of Heidelberg, Heidelberg, Germany); commercially available from CLS (Cell line service, Eppelheim, Germany). Cell panning was performed according to an in-house protocol of the Nanobody Service Facility (VUB, Brussels, Belgium). Frozen NCH cells (5 ×10^6^; NCH644 and NCH421K mix) were thawed on ice and added to 5 mL ice-cold medium of RPMI, 10% fetal calf serum, 2% skimmed milk powder. The cells were mixed with the medium, centrifuged (1500 rpm, 5 min, 4°C), and the supernatant was discarded. The cell pellet was resuspended in 5 mL medium and the mixture was incubated at 4°C for 30 min. In parallel, 10^11^ phages isolated from the library were mixed with medium (total volume, 1 mL) and incubated at 4°C for 30 min, simultaneously with the cells. Next, the cell mixture was centrifuged (1500 rpm, 10 min, 4°C), the supernatant was discarded, and the cell pellet was resuspended in 1 mL phage mixture. This cell–phage mixture was incubated on a rotating platform for 2 h at 4°C, and then transferred to a fresh microcentrifuge tube and centrifuged (2000 rpm, 5 min, 4°C). The supernatant was then removed and the cells were resuspended in 1 mL ice-cold phosphate-buffered saline (PBS), 10% fetal calf serum, 2% skimmed milk, incubated with gentle agitation for 5 min at 4°C, and centrifuged at 2000 rpm for 6 min at 4°C; the supernatant was then removed. The cells were washed in this way four times for the first panning, and seven times for second and third pannings. The cells were then transferred to a new microcentrifuge tube and washed once more with PBS only (twice for second and third pannings). One millilitre of 100 mM triethylamine was added to the cell pellet, and the resulting cell suspension was incubated on a rotating platform for 10 min at 4°C. Then, 1 mL 1 M Tris-HCl was added. Next, 10 mL TG1 cells were infected with 1.9 mL phages, and incubated for 30 min at 37°C without shaking. Then 50 mL 2× yeast extract and tryptone supplemented with 100 μg/mL ampicillin and 2% glucose was added to the TG1 cells, and the suspension was incubated for 30 min at 37°C, with shaking at 225 rpm. After adding 15 μL 10^12^ helper phages/mL, the mixture was incubated for 30 min at 37°C without shaking. The cell mixture was then centrifuged (2000 rpm, 10 min), the supernatant was discarded, and pellet was resuspended in 1 mL 2× yeast extract and tryptone. Then 300 mL 2× yeast extract and tryptone, 100 μg/mL ampicillin, and 70 μg/mL kanamycin was inoculated with the infected TG1 cells and incubated overnight at 37°C with shaking. The next day, the enriched phages were precipitated as described previously [26]. A total of three immunoaffinity enrichment (panning) rounds were performed.

### ELISA screening

Serial dilutions of the phages after the second and third bio-panning were plated on Luria–Bertani–Miller agar plates (Luria–Bertani agar supplemented with 100 μg/mL ampicillin, 1% glucose) and single bacterial clones were used for screening. Nanobodies were selected on the basis of their differential ELISA signal, as described previously [41]. Briefly, 1 mL Terrific broth media was inoculated with a bacterial colony and incubated for 3-4 h at 37°C with shaking (225 rpm). Protein expression was induced by addition of 10 μL 1 M isopropyl β-D-1-thiogalactopyranoside, and the bacterial clones were incubated overnight at 37°C with shaking.

Membrane-protein-enriched fractions of pathological and reference samples were immobilized on ELISA plates (NUNC Maxisorp) at 2 μg/mL (100 μL/well). The plates were placed overnight at 4°C. The next day, the plates were washed three times with 0.1% PBS-Tween and blocked with 200 μL/well 5% PBS-milk, for 1 h at room temperature. The ELISA plates were washed three times with 0.1% PBS-Tween prior to addition of the periplasmic extract.

Bacterial cultures were grown overnight and centrifuged (3220 rpm, 13 min, 4°C), the supernatant was removed and 200 μL Tris/ EDTA/ sucrose was added to each well. After 30 min shaking (150 rpm) at room temperature, 300 μL distilled H_2_O was added to each well, and the shaking was continued for a further 1 h. The cells were then centrifuged (3220 rpm, 13 min, 4°C) and 100 μL of each periplasmic extract was added in parallel to one pathological and one reference sample on the ELISA plate. The samples were incubated for 1 h at room temperature without shaking. The plates were then washed five times with 0.1% PBS-Tween, which was followed by addition of 100 μL/well mouse anti-hemagglutinin (α-HA; 1:2000 dilution) antibody (Sigma Aldrich). There followed an incubation for 1 h at room temperature without shaking. The plates were then washed as before and after the addition of 100 μL/well goat-anti-mouse IgG/ whole molecule/ alkaline phosphatase conjugate (1:2000 dilution; Sigma Aldrich) they were incubated for 1 h at room temperature. Again, the plates were washed five times with 0.1% PBS-Tween and 100 μL/well alkaline phosphatase substrate (Sigma Aldrich) was used for visualization. The intensities of the signals were measured at 405 nm.

### Recloning and production

Nanobody genes for ELISA positive clones were amplified by colony PCR (95°C, 6 min; 94°C, 45 s; 55°C, 45 s; 72°C, 45 s; 72°C, 10 min; 4°C) using the RP (TCA CAC AGG AAA CAG CTA TGA C) and GIII (CCA CAG ACA GCC CTC ATA G) primers, and sequenced at the Genetic Service Facility (VIB, Antwerp, Belgium).

The nanobodies that were chosen for production were amplified with PCR (95°C, 6 min; 94°C, 45 s; 55°C, 45 s; 72°C, 45 s; 72°C, 10 min; 4°C) using the A6E (GAT GTG CAG CTG CAG GAG TCT GGR GGA GG, R=A/G) and 38 (GGA CTA GTG CGG CCG CTG GAG ACG GTG ACC TGG GT) primers. The PCR products were purified and digested overnight with the *Pst*I and *Eco91*I enzymes (Thermo Scientific). The products were ligated for 2 h at room temperature with the pHEN6 vector, which was previously cut with the same enzymes. Then, WK6 *E. coli* cells were transformed with the ligation mixture, plated on Luria–Bertani agar plates, and incubated overnight at 37°C. The bacterial colonies were amplified using the colony PCR described earlier. Nanobodies that had the exact sequence as before recloning with the pHEN6 vector were chosen for large-scale production.

Nanobody production and purification was carried out as described previously [42]. Briefly, single colonies WK6 *E. coli* with the inserted Nb206 sequence were inoculated in 15 mL Luria–Bertani medium supplemented with 100 μg/mL ampicillin, and incubated overnight (37°C, 225 rpm). These overnight cultures were equally distributed in five Erlenmeyer flasks containing 330 mL Terrific Broth medium supplemented with 100 μg/mL ampicillin, 0.1% glucose, 2 mM MgCl_2_ and incubated for 3 h to 4 h at 37°C with shaking at 200 rpm. After addition of isopropyl β-D-1-thiogalactopyranoside to a final concentration of 1 mM, the bacterial cultures were incubated overnight at 28°C with shaking at 200 rpm. The following morning, the bacterial cultures were centrifuged (8000 rpm, 14 min, 4°C) and the supernatant discarded. Pellets were resuspended in 20 mL Tris/ EDTA/ sucrose and incubated for 1 h at 4°C, with shaking at 200 rpm. Then, 40 mL Tris/ EDTA/ sucrose diluted in distilled H_2_O (3:1; v/v) was added and the suspension was incubated for a further 2 h at 4°C, with shaking at 200 rpm. After addition of 500 μL 2 M MgCl_2_, the suspensions were centrifuged (8000 rpm, 30 min, 4°C), and the periplasmic extract was mixed with 5 mL Ni^+^-NTA agarose (Qiagen) and incubated overnight (200 rpm, 4°C). The periplasmic extract with Ni^+^-NTA agarose was loaded into a column and washed with three column volumes of PBS. The expressed nanobodies were eluted with freshly prepared PBS supplemented with 0.5 M imidazole. The nanobodies were then purified using size-exclusion chromatography. Protein concentrations were measured on a Synergy H4 hybrid reader, and brought to a concentration of 1 mg/mL.

### Mass spectrometry and antigen identification

For MS, an antigen:nanobody pair was produced using 1 mg/mL nanobodies and 1.3 mg/mL protein extracted from GSCs. Ni-beads (Protino Ni-TED resin, Macherey–Nagel) were washed and resuspended in PBS, and incubated with and without (control) the nanobodies for 1 h at 4°C on a rotating platform. The Ni-beads were then centrifuged, the supernatants discarded, and 1 mL/tube protein extracted from GSCs was added, with a further incubation with rotation for 1 h at 4°C. The tubes were then centrifuged (12000 rpm, 2 min, 4°C), the supernatants were removed (used for western blot analysis for specific binding of TUFM, Figure 1C), and the pellets washed three times with 1 mL 0.1% PBS-Tween. The pellets were then transferred to fresh tubes and washed again, three times with 1 mL PBS. After centrifugation as before, the supernatant was removed and the antigen:nanobody pair was digested with trypsin solution (2 μL porcine trypsin [Promega] in 100 μL 20 mM triethylammonium hydrogen carbonate buffer), and incubated overnight at room temperature.

After these tryptic digestions, the supernatants were transferred to new microcentrifuge tubes and 1 μL Tris(2-carboxyethyl)phosphine (Thermo Scientific) was added to each tube. Next, 1 μL chloroacetamide was added, and the samples were incubated for 1 h at room temperature. The Ni-beads were then washed once with PBS, the supernatant was discarded, and 25 μL 6 M urea was added to each sample. After centrifugation, the eluted antigens were transferred to new microcentrifuge tubes and the same procedure was repeated twice (i.e., washing with urea and centrifugation). The supernatants were then purified on C18 filters (Empore) [43].

The MS analysis and antigen identification were performed at the Proteomic Facility at the International Centre for Genetic Engineering and Biotechnology in Trieste (Italy). The purified samples were analyzed by liquid chromatography–tandem MS (LC-MS/MS; Easy-nLC system; Bruker) connected to an electron-transfer dissociation ion trap (Amazon; Bruker). The LC was developed using a 75-min gradient from 0% to 80% methanol in 0.1% formic acid. The resulting MS/MS spectra were searched against a human database using the X!tandem and MASCOT search engines, allowing for a 5% false-discovery rate. This led to the identification of one antigen: TUFM.

### Western blotting

Western blotting was performed to monitor the expression of TUFM at the protein level in the different GBM samples. The proteins (20 μg) were denatured in NuPAGE LDS Sample Buffer (4x), and loaded onto a NuPAGE 4% - 12% Bis-Tris gel. The separated proteins were transferred onto polyvinyldidene fluoride (PVDF) membranes. The total protein was stained using Ponceau S. The PVDF membranes were blocked for 1 h at room temperature in PBS containing 5% non-fat dried milk, and incubated overnight at 4°C in 1% PBS-milk containing the primary antibody (mouse anti-TUFM antibody, 1:1000 dilution; Sigma-Aldrich). The membranes were washed in 0.1% PBS-Tween, incubated with the secondary antibody (anti-mouse IgG HRP-conjugated antibody; 1:5000 dilution; Jackson ImmunoResearch) for 1 h at 4°C, and washed in 0.1% PBS-Tween. Membrane was treated with SuperSignal West Femto Chemiluminescent Substrate (Thermo Scientific) and the bands were visualized using a LAS-4000 CCD camera (Fujifilm; Tokyo, Japan). The bands were analyzed with the Multi Gauge version 3.2 software.

### RT-qPCR

Messenger RNA was extracted from 13 glioblastomas and 10 paired normal samples (hippocampus, periventricular and subventricular zone of 10 post-mortem brain samples), two GBM cell lines (U87MG, U251MG), GSCs and NSCs using the TRI reagent (Sigma Aldrich), as described by the manufacturer. The concentrations and purities of the extracted mRNAs were determined (NanoDrop ND-1000; NanoDrop Technologies, USA) and the RNA integrity was examined (Agilent 2100 bioanalyser; Agilent Technologies, USA). For reverse transcription to cDNA, 3 μg mRNA was used. The samples were first treated with recombinant RNAse free DNase I (Roche) for 15 min at 30°C and for 10 min at 75°C, and then transcribed (Transcriptor Universal cDNA Master; Roche) for 5 min at 25°C, 10 min at 55°C, and 5 min at 85°C.

Five reference genes were tested on a pooled sample with 2× LightCycler 480 SYBR Green I Master (Roche) on a LightCycler 480 system (Roche): *TBP, HPRT1, RPL13A, GAPDH* and *CYC1* [44–46]. The qPCR primers for the reference genes were chosen from the literature [44] and are given in Table 2. The efficiencies of the primer pairs were determined using the standard curve method with pooled cDNA serial dilutions. The stabilities of the genes were determined using NormFinder.

The primers for the genes under study were acquired from the PrimerBank database (https://pga.mgh.harvard.edu/primerbank/) and are given in Table 2. The samples were analyzed using 3 μL cDNA in 20 μL reaction mixtures with 2× LightCycler 480 SYBR Green I Master (Roche) and 5 μM primers. The samples were tested in triplicates using the following protocol: pre-incubation: 10 s, 95°C; cycling: 20 s, 60°C; 20 s, 72°C; for 45 cycles; melting curve: 5 s, 95°C; 1 min, 65°C; continuous 97°C; cooling: 30 s, 40°C. The study genes were normalized to the reference genes. Group comparison by t-test was performed in GraphPad Prism 6 for the tissue samples. Multiple group comparisons using one way ANOVA were performed in GraphPad Prism 6 for the cell lines.

### Cytotoxicity measurements

The numbers of viable cells were determined using the WST-1 (2-(4-iodophenyl)-3-(4-nitrophenyl)-5-(2.4-disulfophenyl)-2H-tetrazolium, sodium salt; Roche) colorimetric assay. Here, 4 ×10^3^ cells were seeded in 100 μL medium in triplicates using 96-well flat-bottomed plates, and incubated for 2 h at 37°C. The cultures were incubated with Nb206 at two different concentrations (10 μg/mL, 100 μg/mL) for 24 h and 48 h. Controls were treated as the GBM and control cells, but without the addition of Nb206. After these incubations, 10 μL WST-1 reagent was added to each well, which were then incubated for 2 h at 37°C. The absorbances were measured at 450 nm against a reference wavelength of 620 nm using a microtiter plate reader (BioTek, Synergy H4). The blank was the absorbance of the culture media without the cells.

### Apoptosis and necrosis of glioblastoma cell lines and glioblastoma stem cells

For the apoptosis/necrosis tests we used a commercially available kit Annexin V-FITC conjugated/ Propidium Iodide (Abcam) according to the manufacturer’s instructions. Glioblastoma cell lines U251MG and U87MG were grown on glass cover slips (250,000 cells), while glioblastoma stem cells (500,000) were grown in suspension and then transferred onto a cover slip. After 6 h of growth they were incubated with Nb206 for 16 h at 37°C. Controls were treated the same, except without addition of Nb206. Green and red signals were observed by fluorescent microscopy (Axio Imager M2, Zeiss) with the images handled using the ZEN software.

### Visualization of three-dimensional interactions between Nb206 and TUFM

With the *in-silico* analysis, among several targets of the nanobodies tested, Nb206 (this study) and Nb225 (our previous study) [30] were seen to interact with the human mitochondrial elongation factor EF-TU (i.e., TUFM). As the 3D structures of these nanobodies had not been solved, suitable homology building templates were searched for. Nb206 and a camelid nanobody (4WGV) showed high sequence identity (76%), with only one single amino acid deletion (a missing serine after T104), as related to the solved protein-protein complex for PDB entry 4WGV [27]. The ALIGN and BLDPIR commands in the WHATIF modelling suite [28] were used for homology building of Nb206 with this template. After manual deletion of the corresponding serine, several short optimization runs were performed with the CHARMM molecular simulation program [47], and with structure correction using the DGLOOP subset of commands in WHATIF. When a good quality score was achieved with the PROCHECK module in WHATIF, the 3D model of Nb206 was submitted to a protein–protein docking suite together with the structure of human EF-TU (PDB code 1D2E), as implemented with the Rosetta online server (http://rosie.rosettacommons.org) [48]. The initial positions of Nb206 and EF-TU were generated on the basis of PROBIS analysis of protein binding sites [49], which revealed that the N-terminal surface of Nb206 is a principal interaction surface for the target antigens. Subsequently, the best resulting complex structure was put in a cube of 27,514 water molecules, together with 79 sodium and 77 chloride ions for electrical neutrality, and was subjected to 150 relaxation steps (50 steps of s.d. optimization; 50 steps of optimization by the adopted basis of the Newton-Raphson method; 50 steps of descent lattice optimization), followed by 400 ns constant pressure and temperature dynamic simulation (300 K; 1 bar; time step, 1 fs). The EWALD summation was used for calculation of the electrostatic interactions, with the aim being to check the stability and correctness of the complex. Furthermore, an analogous molecular simulation of 200 ns was run with the opposite interaction surface of Nb206 to compare its stability with the first orientation.

### Immunocytochemistry

Immunocytochemistry was performed using the specific U87MG and U251MG GBM cell lines, and GSCs, to determine the specificity of Nb206 towards its antigen. These cells (100,000 cells) were attached to glass cover slips pretreated with poly-D-lysine (Sigma-Aldrich). The attached cells were fixed with ice-cold 4% paraformaldehyde for 15 min and then permeabilized with PBS/0.1% Triton X-100 for 15 min. After a 1-h incubation at room temperature in blocking buffer (1% bovine serum albumin in PBS), the cells were incubated overnight at 4°C with a mouse anti-TUFM antibody (1:1000 dilution; Sigma-Aldrich) or with Nb206 FITC conjugated. Following four 10-min washes with PBS, the cells incubated with the commercial primary antibodies were incubated with secondary antibodies goat anti-mouse IgG (1:3000 dilution; Sigma-Aldrich). The incubation with the secondary antibodies was performed at room temperature for 1.5 h. The cells were then washed four times for 10 min each with PBS, and stained with 50 μL 300 nM DAPI for 10 min. The cells were then washed again for four times for 10 min with PBS, and mounted on slides with Hydromount (National Diagnostics). The cells were examined under a fluorescence microscope (TE2000-E; Nikon Eclipse, Japan), with the images handled using the ImageJ software.

To confirm the colocalization of Nb206 with mitochondria in living cells, the U251MG cell line was used. The cells (50,000) were attached on glass cover slips and incubated with Nb206 for 24h at 37°C. The cells were then treated with Hoechst 33342 (Thermo Fischer Scientific), diluted 1:2000 for 10 min at room temperature in the dark, washed three times with PBS and treated with 50 nM MitoTracker Orange CMTMRos for 15 min in the dark and at room temperature. The cells were then washed three times with PBS and fixed with ice-cold 4% paraformaldehyde for 10 min, washed again with PBS and mounted on slides with Hydromount (National Diagnostics). The cells were observed under an inverted confocal laser scanning microscope (Axio Observer Z1 LSM 710, Zeiss, Germany) and the images analyzed using the ZEN software (Zeiss, Germany).

### Statistical analysis

All of the experiments were performed at least three times. The data were analyzed for statistical significance using one-way ANOVA followed by Sidak`s and Dunnett`s multiple comparison tests. P values <0.05 were considered to indicate statistical significance. The means ± standard deviation were calculated for all of the variables.

## SUPPLEMENTARY MATERIALS FIGURES AND TABLE





## Abbreviations

3D: three-dimensional
A_260_/A_280_: 260 nm to 280 nm absorption ratio
AU: arbitrary units
ELISA: enzyme-linked immunosorbent assay
EMT: epithelial-to-mesenchymal transition
GBM: glioblastoma multiforme
GSCs: glioblastoma stem cells
MS: mass spectrometry
Nb206: anti-TUFM nanobody
PBS: phosphate-buffered saline
TUFM: mitochondrial translation elongation factor (EF-TU)
VHH: variable domain of a heavy chain
