# Supplementary material for: Glioblastoma-specific anti-TUFM nanobody for in-vitro immunoimaging and cancer stem cell targeting
Source: Oncotarget. 2018 Apr 3;9(25):17282–99. doi: 10.18632/oncotarget.24629 (PMC5915116; doi:10.18632/oncotarget.24629)
Supplement: Supplementary file 1 [file oncotarget-09-17282-s001.pdf]

Glioblastoma-specific anti-TUFM nanobody for *in-vitro* immunoimaging and cancer stem cell targeting

SUPPLEMENTARY MATERIALS

Supplementary Table 1: ELISA signals for glioblastoma stem cell (GSC in rows A,C,E,G) and normal tissue lysate (NTL in rows B,D,F,H) from 3 different cell pannings. CPC1 – 1st panning on whole GSC cells, CPC2 – 2nd panning on whole GSC cells and CPC3 – 3rd panning on whole GSC. With red and bold are marked 1.9-fold higher ELISA signals in GSC than in normal tissue lysates. Signal for Nb206 is additionally highlighted with yellow.

See Supplementary File 1

A

ENSP00000322439: TUFM:p, Tu translation elongation factor, mitochondrial
log(e) = -23.3 [Source:HGNC Symbol;Acc:HGNC:12420]


```
  1 MTTMAAATLLRATPHFSGLAAGRTFLLQGLLRLLKAPALPLLCRGLAVEAKKTYVRDKPH  60
 61 VNVGTIGHVDHGKTTLTAAITKILAEGGGAKFKKYEEIDNAPEERARGITINAAHVEYST 120
121 AARHYAHTDCPGHADYVKNMITGTAPLDGCILVVAANDGPMPQTREHLLLARQIGVEHVV 180
181 VYVNKADAVQDSEMVELVELEIRELLTEFGYKGEETPVIVGSALCALEGRDPELGLKSVQ 240
241 KLLDAVDTYIPVPARDLEKPFLLPVEAVYSVPGRGTVVTGTLERGILKKGDECELLGHSK 300
301 NIRTVVTGIEMFHKSLERAEAGDNLGALVRGLKREDLRRGLVMVKPGSIKPHQKVEAQVY 360
361 ILSKEEGGRHKPFVSHFMPVMFSLTWDMACRIILPPEKELAMPGEDLKFNLILRQPMILE 420
421 KGQRFTLRDGNRTIGTGLVTNTLAMTEEEKNIKWG                          455
```

show legend ?

MVDQP upper case sequence is the protein sequence originally analyzed

DIMR residues part of at least one observed peptide domain

LREEQ residues predicted to be difficult to observe by standard techniques

HFQL residue found is a single amino-acid polymorphism

AYNG residue found is chemically modified

Complete mods: i. Carbamidomethyl@C

Potential mods: i. Oxidation@M
ii. Oxidation@M, Oxidation@W, Deamidated@N, Deamidated@Q
iii. Dioxidation@M, Dioxidation@W

Protein-specific PTMs: i. Acetyl@K

N-terminal: i. Ammonia-loss@Q, Ammonia-loss@C, Dehydrated@E (peptide)
ii. ragged, Acetyl (protein)

Identified Peptides

spectrum	log(e)	log(I)	m+h	delta	ζ	sequence	n
1257.1	-11.3	7.27	2129.164	-0.017	2/3	vpar256 DLEKPFLLPV EAVYSVPGR 274gtvv	(54633)
1264.1	-1.3	6.49	2129.164	-0.101	2/3	vpar256 DLEKPFLLPV EAVYSVPGR 274gtvv	(54633)
906.1	-7.3	7.77	1185.622	-0.094	2/2	sler319 AEAGDNLGAL VR 330glkr	(67443)

B

#	log(e)	log(I)	m+h	delta	ζ	sequence validate | studio | mgf | mrm | details
1257	-11.3	7.27	2129.164	-0.017	2/3	vpar256 DLEKPFLLPVEAVYSVPGR274gtvv (54633) 0.031

Cmpd 1597, +MSn(1065.0770), 19.4 min

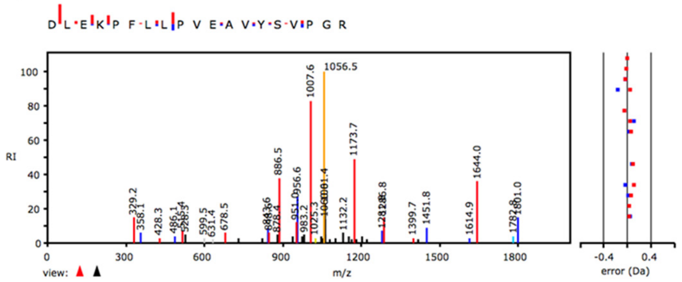

matched/total: # ions: 50% intensity: 80% μ: 0.02, σ: 0.06 Da

bond	+1y	+1y-17	+1y-18	+1b	+1b-17	+1b-18
D1	+2 2014.137	1997.111	1996.127	116.034	99.008	98.024
L2	+2 1901.053	1884.027	1883.043	229.118	212.092	211.108
E3	+2 1772.011	1754.984	1754.000	358.161	341.134	340.150
K4	1643.916	1626.889	1625.905	486.256	469.229	468.245
P5	1546.863	1529.836	1528.852	583.309	566.282	565.298
F6	1399.794	1382.768	1381.784	730.377	713.350	712.366
L7	1286.710	1269.684	1268.700	843.461	826.435	825.451
L8	1173.626	1156.600	1155.616	956.545	939.519	938.535
P9	1076.573	1059.547	1058.563	1053.598	1036.571	1035.587
V10	977.505	960.479	959.495	1152.666	1135.640	1134.656
E11	848.462	831.436	830.452	1281.709	1264.682	1263.698
A12	777.425	760.399	759.415	1352.746	1335.719	1334.735
V13	678.357	661.330	660.346	1451.814	1434.788	1433.804
Y14	515.294	498.267	497.283	1614.878	1597.851	1596.867
S15	428.262	411.235	410.251	1701.910	1684.883	1683.899
V16	329.193	312.167	311.183	1800.978	1783.952	1782.968
P17	232.140	215.114	214.130	1898.031	1881.004	1880.020
G18	175.119	158.092	157.108	1955.052	1938.026	1937.042

Supplementary Figure 1: (A) Mass spectrometry identifies TUFM as a nanobody target. The sequence of TUFM is shown with the matched peptides highlighted red and the green residues indicate areas that are predicted to have poor coverage in the mass spectrometer. The match statistics for the matching peptides are shown below. **(B)** Interpreted MS/MS spectrum from TUFM. The interpreted spectra for the best matching peptide from TUFM is shown. Matched peaks are shown in color and unmatched peaks in black. The match table for the b- and y- ions is shown below the spectrum and the distribution of mass errors is shown to the right of the spectra.

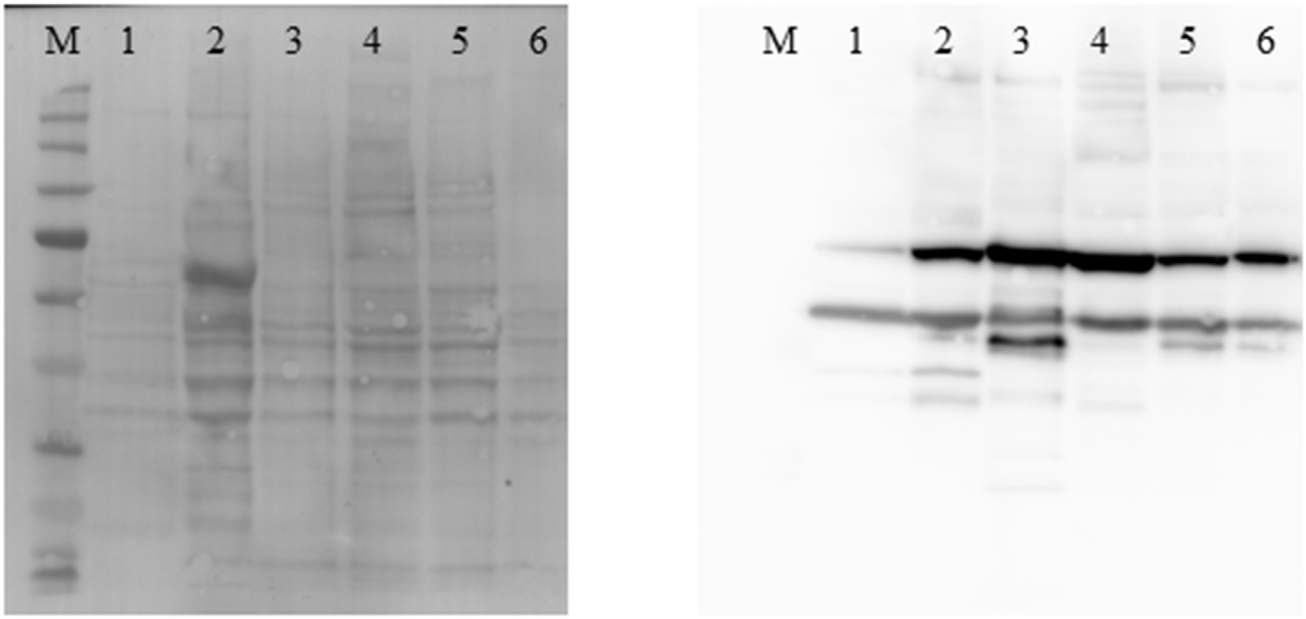

Supplementary Figure 2: Left. Western blot membrane stained with PonceauS. Right. Western blot membrane for TUFM (43 kDa) and GAPDH (35 kDa). Sample order: M - Blue Star Prestained Protein Marker; 1 - Normal brain tissue (REFt), 2- glioblastoma tissue (GBMt), 3 - neural stem cells (NSC), 4 - glioblastoma stem cells (GSC), 5 - glioblastoma cell line U251MG and 6 – glioblastoma cell line U87MG.
